# Persistent Median Artery Thrombosis Presenting as a Mobile Volar Hand Mass: A Rare Case With Surgical Considerations

**DOI:** 10.1002/ccr3.73177

**Published:** 2026-07-20

**Authors:** Elise H. Katsnelson, Jochen Gerstner Saucedo, Zachary Stauber, Olivia B. de Araujo, Helen G. Hui‐Chou, Jean Jose

**Affiliations:** ^1^ University of Miami Miller School of Medicine Miami Florida USA; ^2^ Department of Radiology, Children's Hospital Colorado University of Colorado Anschutz Medical Campus Aurora Colorado USA; ^3^ Department of Orthopaedic Surgery University of Miami Miller School of Medicine, Jackson Memorial Hospital Miami Florida USA; ^4^ Department of Plastic Surgery University of Miami Miller School of Medicine, Jackson Memorial Hospital Miami Florida USA; ^5^ Department of Radiology University of Miami Miller School of Medicine, Jackson Memorial Hospital Miami Florida USA

**Keywords:** orthopedics, Radiology & Imaging, surgery, vascular surgery

## Abstract

Persistent median artery thrombosis can present atypically as a painless, mobile volar hand mass that mimics a benign lesion. Awareness of this vascular variant and use of preoperative imaging are essential for accurate diagnosis, while vein graft reconstruction offers a safe approach to preserve arterial continuity and hand perfusion.

## 1 Introduction

The persistent median artery (PMA) is a developmental variant that arises from the failure of median artery regression during embryonic development, with a reported prevalence of approximately 20%–43% per limb in the general population [1]. Embryologically, the PMA originates from the axial artery of the developing limb bud and typically regresses as the radial and ulnar arteries become the dominant blood supply to the hand [2, 3]. When this regression fails, the vessel may persist with variable origins and anatomical relationships to the median nerve [4, 5].

Clinically, the PMA is often asymptomatic but is of surgical importance due to its proximity to the median nerve, especially within the carpal tunnel [2, 5, 6]. It may contribute to the superficial palmar arch and hand perfusion and, when thrombosed, can lead to acute or chronic carpal tunnel syndrome, pseudoaneurysm formation, or ischemic symptoms [3, 7, 8]. The PMA may also penetrate or divide the median nerve, increasing the risk of compression, and complicating surgical decompression [4, 5].

While some studies suggest an association with carpal tunnel syndrome, its exact role remains debated [9, 10]. Preoperative imaging with ultrasound or MRI is recommended to identify the PMA and its variations to minimize surgical complications [6, 9]. Despite the relatively common prevalence of a persistent median artery (~20%–43% per limb), the combination of a painless presentation, pseudoaneurysm formation, and venous graft reconstruction is rarely reported [1, 11, 12, 13, 14, 15, 16]. A comparative overview of previously reported cases is shown in Table 1. Herein, we present the case of a 41‐year‐old female with a thrombosed persistent median artery presenting as a painless, mobile volar hand mass, managed with pseudoaneurysm resection and vein graft reconstruction.

**TABLE 1:** Comparative overview of previously reported cases of persistent median artery thrombosis.

Study	Age/Sex	Presentation	Bifid Nerve	Treatment	Outcome
Levy and Pauker (1978) [6]	72/F	Acute CTS	Not reported	CTR + artery left in situ	Symptom resolution
Barfred et al. (1985) [2]	Multiple	CTS symptoms	Yes	CTR	Symptom improvement
Dickinson and Kleinert (1991) [7]	46/M	Acute CTS, calcified PMA	Not reported	CTR + excision of calcified segment	Complete resolution
Bilgin et al. (2004) [9]	Multiple	CTS (4 patients)	Variable	Simple CTR	Resolution in 2/3; 1 recurrence
Salter et al. (2011) [4]	42/F	Acute CTS	Yes	CTR + thrombectomy	Complete resolution
Srivastava et al. (2015) [3]	38/M	Subacute CTS	Not reported	Conservative	Symptom resolution
Arnauw and De Wachter (2021) [14]	39/M	CTS, repetitive wrist use	Not reported	CTR + excision	Symptom resolution
Barr et al. (2022) [5]	41/F	Acute CTS	Yes	CTR + excision of thrombosed PMA	Resolution at 3 months
Sheridan et al. (2022) [15]	29/M	Acute CTS, professional hockey player	Not reported	CTR + surgical excision of thrombosed PMA	Return to sport
Horan et al. (2023) [8]	20/F	Acute CTS post‐vaccination	Not reported	CTR + ligation/excision	Symptom resolution
Baskar et al. (2025) [16]	29/F	Acute CTS	Yes	Anticoagulation + anti‐edema	Resolution at 2 weeks
Present case	41/F	Painless mobile volar mass	No	Pseudoaneurysm resection + vein graft	Complete resolution at 3 months

Abbreviations: CTR, carpal tunnel release; CTS, carpal tunnel syndrome; PMA, persistent median artery.

## 2 Case History/Examination

**FIGURE 1:**
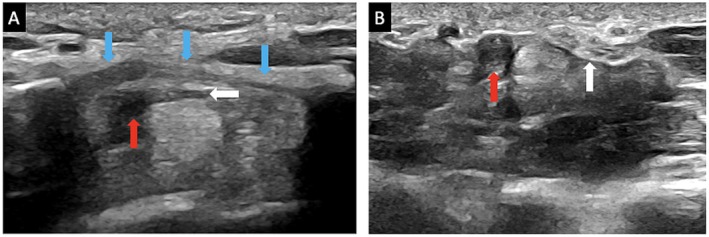
Sequential transverse gray‐scale ultrasound images showing persistent median artery pseudoaneurysm with thrombosis at (A) and just distal (B) to the level of the carpal tunnel. Images demonstrate a hypoechoic tubular mass (red arrows) adjacent to the compressed median nerve (white arrows), flexor tendons, and transverse carpal ligament (blue arrows).

**FIGURE 2:**
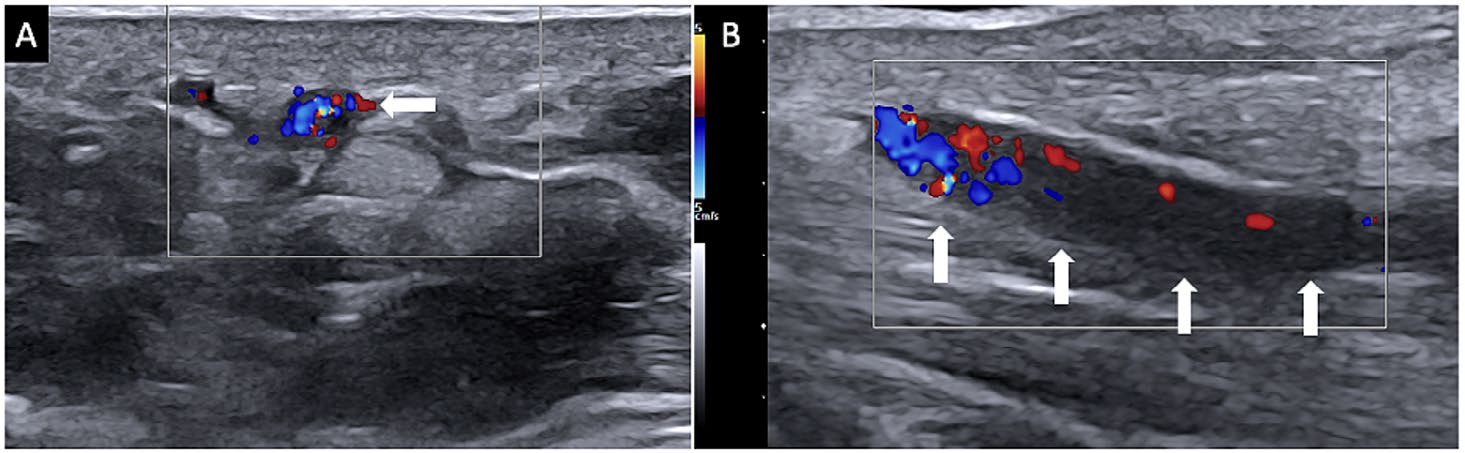
Transverse (A) and longitudinal (B) gray‐scale ultrasound images demonstrate a dilated hypoechoic tubular mass with internal echogenic material and lack of normal arterial flow at the level of the carpal tunnel (white arrows).

A 41‐year‐old female presented with a two‐month history of a mobile, painless volar hand mass. Physical examination revealed a 0.5 × 2 cm mobile, non‐erythematous, nonpulsatile, non‐tender mass with intact capillary refill and normal sensation. MRI demonstrated a 5 mm round hyperintense tubular mass within the carpal tunnel. At the same time, ultrasound confirmed a persistent median artery with pseudoaneurysm formation and internal thrombosis at and distal to the carpal tunnel measuring approximately 2 cm (Figure 1), with a lack of arterial flow noted on Doppler (Figure 2).

## 3 Differential Diagnosis, Investigations and Treatment

The painless, mobile nature of the lesion initially suggested a benign soft‐tissue mass such as a ganglion cyst or lipoma. However, the location within the carpal tunnel and the vascular characteristics identified on imaging helped narrow the diagnosis to a thrombosed persistent median artery with pseudoaneurysm formation.

Ultrasound revealed a hypoechoic tubular mass with internal echogenic material and no arterial flow, consistent with a thrombosed vessel (Figures 1 and 2), while MRI showed a hyperintense tubular lesion confirming its vascular origin. These imaging findings were essential in distinguishing the lesion from non‐vascular soft‐tissue masses and confirming the diagnosis prior to surgical planning. Due to concerns for emboli or eventual median nerve compression from an already present thrombus, surgical treatment options were discussed with the patient, including carpal tunnel exploration with ligation and excision of the pseudoaneurysm, pseudoaneurysm resection with vein graft reconstruction of the persistent median artery, and pseudoaneurysm resection with ipsilateral lateral circumflex femoral artery reconstruction of the persistent median artery. After consideration of treatment options, the patient elected for surgical resection of the pseudoaneurysm with vein graft reconstruction of the persistent median artery, which was performed within a week. The decision for vein graft reconstruction over simple ligation was supported by the following considerations: (1) the patient's age (41 years) and active lifestyle warranted preservation of arterial redundancy; (2) intraoperative Doppler confirmed adequate digital perfusion with temporary clamping, but the PMA's potential contribution to the superficial palmar arch could not be definitively excluded; (3) the risk of a blind arterial stump within the carpal tunnel potentially causing recurrent thrombosis or nerve irritation; and (4) the 2 cm thrombosed segment with pseudoaneurysm formation posed an ongoing embolic risk if left in situ [2, 9].

**FIGURE 3:**
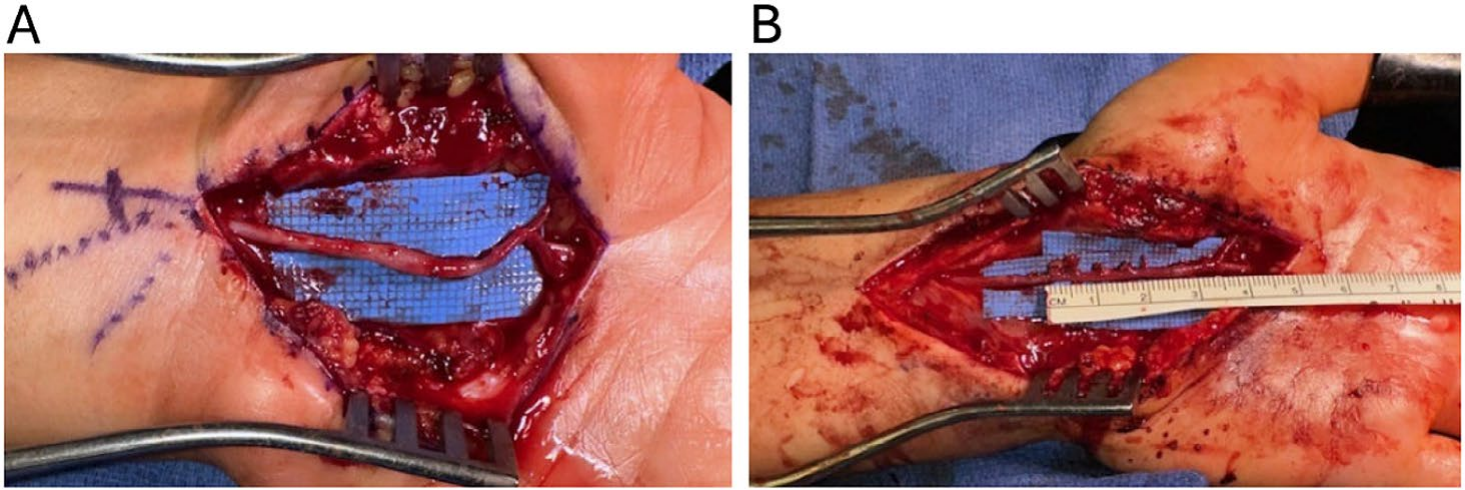
Intraoperative images of thrombosed median artery before (A) and after (B) vein graft reconstruction.

**FIGURE 4:**
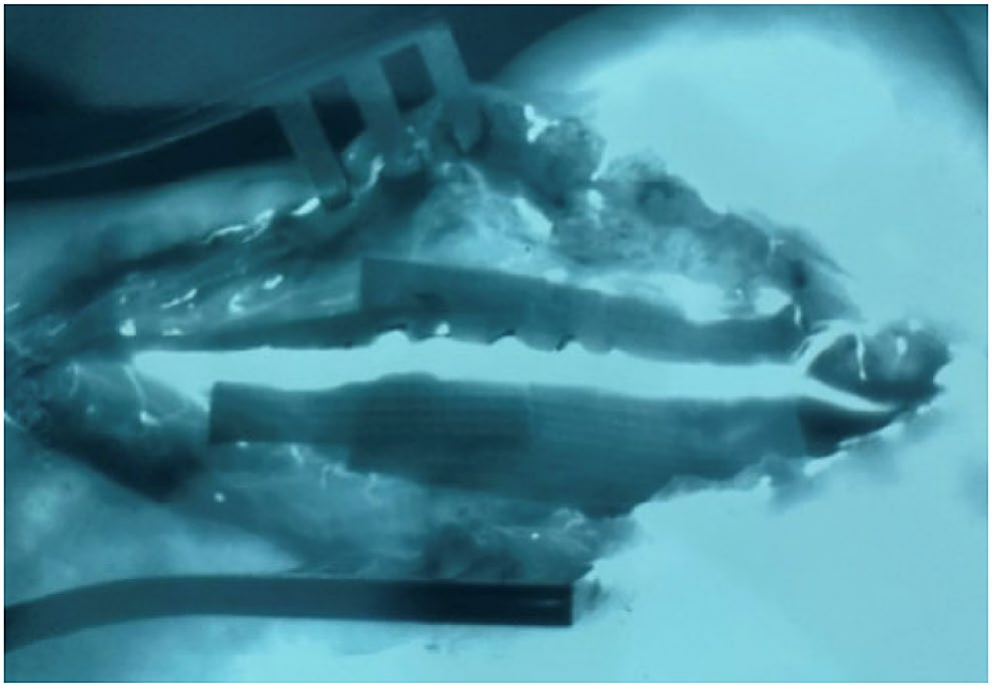
Intraoperative laser angiography of the median artery after graft reconstruction.

Intraoperative assessment identified the thrombosed persistent median artery under the transverse carpal ligament, and complete neurolysis of the median nerve was then carried out. The artery was clamped distal to its thrombosis, and flow to all digits was confirmed with Doppler prior to resection of the thrombotic segment and reconstruction with a reverse vein graft (Figure 3). At the conclusion of the case, flow was confirmed via intraoperative Spy Phi Laser angiography (Figure 4).

This study is a single‐patient case report and did not require institutional review board approval. Ethical oversight was waived in accordance with institutional policies for case reports. All procedures followed the principles outlined in the Declaration of Helsinki.

## 4 Conclusion and Results

**FIGURE 5:**
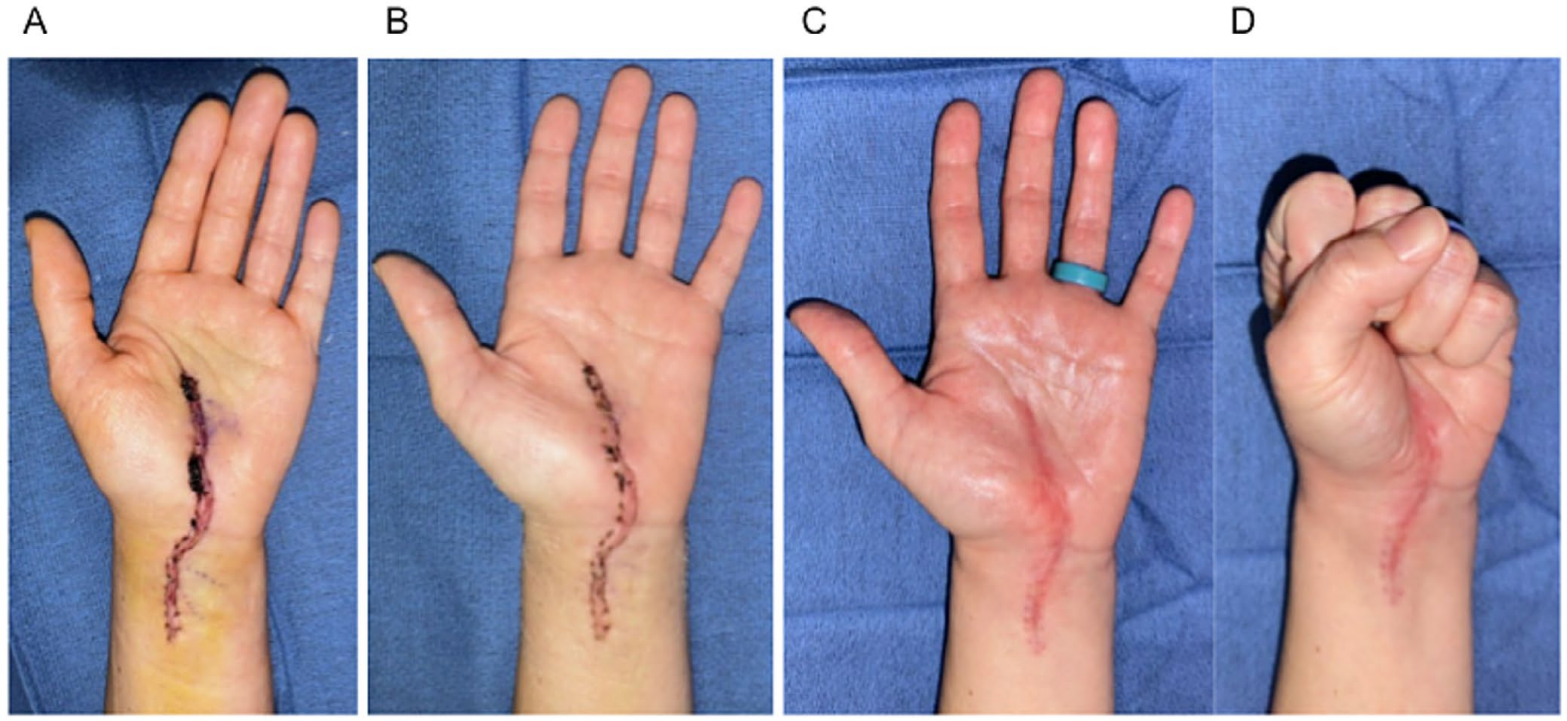
Postoperative clinical photographs demonstrating wound healing and functional recovery. (A) Five days, (B) 17 days, and (C, D) three months postoperatively, demonstrating progressive wound healing with a well‐healing palmar scar and full range of motion at final follow‐up.

Postoperatively, the patient recovered well without complications. At her latest follow‐up three months after the procedure, the patient noted complete symptom resolution, and the clinical exam showed full range of motion, no signs of inadequate perfusion, and a well‐healing palmar scar (Figure 5). At the five‐day, 17‐day, and three‐month follow‐up visits, vascular examination demonstrated intact radial and ulnar pulses, a palpable graft pulse, and brisk capillary refill in all digits. No recurrence of the volar mass or new neurological symptoms was noted.

Persistent median artery thrombosis is a rare but essential consideration in the evaluation of volar hand masses, particularly when lesions are mobile, painless, and mimic benign soft‐tissue tumors. Although often asymptomatic, a thrombosed PMA can pose a risk for median nerve compression or distal embolization if left untreated. Early recognition through imaging, especially with ultrasound and MRI, is essential for accurate diagnosis. Surgical management should be guided by vascular anatomy and perfusion considerations, with vein graft reconstruction offering a safe and effective option in select cases. Increased awareness of this entity can help prevent delays in diagnosis and optimize patient outcomes.

## 5 Discussion

Though typically regressing during early gestation, the median artery can persist and pass through the carpal tunnel, causing pain in adulthood from either its native anatomy or enlargement secondary to pseudoaneurysm and thrombosis [3]. Several cases [3, 4, 5, 8, 17] have reported thrombosis of a persistent median artery presenting as a forearm mass. However, our case is distinct in that the thrombosis manifested as a painless, slowly shrinking, and mobile volar mass rather than with typical ischemic or compressive symptoms.

The etiology of persistent median artery thrombosis is multifactorial and remains incompletely understood. Proposed mechanisms include repetitive mechanical trauma from wrist movements (particularly flexion and extension), external compression within the confined space of the carpal tunnel, hormonal factors such as oral contraceptive use, local infection of deep fascial planes, and endothelial injury secondary to vibration exposure [3, 6, 8, 16]. The relatively small caliber of the PMA and its passage through the carpal tunnel may predispose it to flow stasis and subsequent thrombosis, particularly during repetitive wrist use. In our patient, no clear precipitating factor was identified; however, the absence of systemic thrombophilia risk factors and the localized nature of the thrombus suggest a mechanical or anatomical etiology. Recent reports have also raised a possible association between COVID‐19 vaccination and PMA thrombosis, though a causal relationship has not been established [8, 14, 15, 16]. The idiopathic nature of thrombosis in this case further underscores the importance of considering PMA thrombosis in the differential diagnosis of volar hand masses even in the absence of identifiable risk factors.

Surgical decision‐making involved consideration of resection with vein graft, simple ligation, or an arterial graft from the lateral circumflex femoral artery. Although duplex demonstrated absent flow within the PMA, we elected resection with a reverse‐vein graft reconstruction to remove the diseased segment and pseudoaneurysm, preserve potential contribution to the superficial palmar arch in the event of distal recanalization or changing collateral dynamics, avoid the creation of a blind arterial stump within the carpal tunnel that could thrombose or irritate the median nerve, and maintain arterial redundancy in a younger, active patient. Adequate digital perfusion with temporary clamping was confirmed intraoperatively prior to reconstruction. Prior reports caution against simple excision when the PMA contribution is uncertain [2, 9].

Excision of the artery is generally dissuaded out of concern for loss of perfusion to the digits [2, 9]. Ultrasound and MRI played a critical role in characterizing the lesion, distinguishing it from soft‐tissue tumors, and identifying the thrombosed PMA. Preoperative imaging should be considered essential in evaluating atypical volar hand masses. Ultimately, the patient experienced excellent recovery after resection with vein grafting. While three‐month follow‐up demonstrated complete symptom resolution, intact graft perfusion, and full range of motion (representing a meaningful clinical endpoint), extended follow‐up would further confirm the long‐term durability of the reconstruction.

Given the ~20%–43% limb prevalence of a persistent median artery, our patient's painless pseudoaneurysm with thrombosis and successful venous graft reconstruction underscores an uncommon presentation that supports individualized vascular planning [1, 11, 12, 13, 18]. This case highlights the need to consider vascular anomalies not only when assessing carpal tunnel syndrome, but also when making a differential for volar hand masses that mimic benign lesions such as ganglion cysts or lipomas. It underscores the value of detailed preoperative imaging, anatomical awareness, and individualized surgical planning in achieving optimal outcomes.

## Author Contributions

**Elise H. Katsnelson:** conceptualization, data curation, formal analysis, methodology, project administration, writing – original draft, writing – review and editing. **Jochen Gerstner Saucedo:** conceptualization, data curation, formal analysis, investigation, methodology, project administration, writing – original draft, writing – review and editing. **Zachary Stauber:** conceptualization, data curation, formal analysis, methodology, project administration, writing – original draft, writing – review and editing. **Olivia B. de Araujo:** conceptualization, data curation, formal analysis, methodology, project administration, writing – original draft, writing – review and editing. **Helen G. Hui‐Chou:** conceptualization, data curation, formal analysis, investigation, methodology, project administration, supervision, validation, writing – review and editing. **Jean Jose:** conceptualization, data curation, investigation, methodology, project administration, resources, supervision, writing – review and editing.

## Funding

The authors have nothing to report.

## Ethics Statement

All procedures followed were in accordance with the ethical standards of the responsible committee on human experimentation (institutional and national) and with the Helsinki declaration of 1975, as revised in 2008 (5). Informed consent was obtained from all patients for being included in the study.

## Consent

Written informed consent was obtained from the individual participant regarding the surgical procedures performed, though no identifying information is included in this case report.

## Conflicts of Interest

The authors declare no conflicts of interest.

## Data Availability

Data sharing not applicable to this article as no datasets were generated or analyzed during the current study.

## References

[1] C. Ellis , D. Thibault , J. Lencke , and L. D. Hemric , “Prevalence and Anatomical Significance of the Persistent Median Artery: A Cadaveric Study,” PLoS One 20 (2025): e0320288, 10.1371/journal.pone.0320288. 40163473 PMC11957254

[2] T. Barfred , A. P. Højlund , and K. Bertheussen , “Median Artery in Carpal Tunnel Syndrome,” Journal of Hand Surgery 10 (1985): 864–867, 10.1016/s0363-5023(85)80163-5. 4078270

[3] A. Srivastava , P. Sharma , and S. Pillay , “Persistent Median Artery Thrombosis: A Rare Cause of Carpal Tunnel Syndrome,” Australas J Ultrasound Med 18 (2015): 82–85, 10.1002/j.2205-0140.2015.tb00047.x. 28191246 PMC5024968

[4] M. Salter , N. R. Sinha , and W. Szmigielski , “Thrombosed Persistent Median Artery Causing Carpal Tunnel Syndrome Associated With Bifurcated Median Nerve: A Case Report,” Polish Journal of Radiology 76 (2011): 46–48. 22802832 PMC3389914

[5] M. L. Barr , N. S. Jain , P. A. Ghareeb , and P. Benhaim , “Persistent Median Artery Thrombosis Causing a Bifid Median Nerve and Carpal Tunnel Syndrome: A Case Report,” JBJS Case Connector 12, no. 4 (2022), 10.2106/JBJS.CC.22.00424. 36206366

[6] M. Levy and M. Pauker , “Carpal Tunnel Syndrome Due To Thrombosed Persisting Median Artery. A Case Report,” Hand 10 (1978): 65–68, 10.1016/s0072-968x(78)80028-x. 710985

[7] J. C. Dickinson and J. M. Kleinert , “Acute Carpal‐Tunnel Syndrome Caused by a Calcified Median Artery. A Case Report,” Journal of Bone and Joint Surgery. American Volume 73 (1991): 610–611, 10.2106/00004623-199173040-00020. 2013602

[8] E. Horan , P. Romeo , and A. Loch‐Wilkinson , “Carpal Tunnel Syndrome Caused by Persistent Median Artery Thrombosis With a Possible Link to COVID‐19 Vaccination: A Case Report,” Australasian Journal of Plastic Surgery 6 (2023): 1–3, 10.34239/ajops.v6n1.70244.

[9] S. S. Bilgin , S. E. Olcay , A. Derincek , S. Adiyaman , and A. M. Demirtas , “Can Simple Release Relieve Symptoms of Carpal Tunnel Syndrome Caused by a Persistent Median Artery? Clinical Experience,” Archives of Orthopaedic and Trauma Surgery 124 (2004): 154–156, 10.1007/s00402-004-0637-x. 14767781

[10] W. Luyendijk , “The Carpal Tunnel Syndrome,” Acta Neurochirurgica 79 (1986): 52–57, 10.1007/bf01403466. 3953326

[11] B. Solewski , M. Lis , J. R. Pękala , et al., “The Persistent Median Artery and Its Vascular Patterns: A Meta‐Analysis of 10,394 Subjects,” Clinical Anatomy 34 (2021): 1173–1185, 10.1002/ca.23770. 34371525

[12] T. Lucas , J. Kumaratilake , and M. Henneberg , “Recently Increased Prevalence of the Human Median Artery of the Forearm: A Microevolutionary Change,” Journal of Anatomy 237 (2020): 623–631, 10.1111/joa.13224. 32914433 PMC7495300

[13] P. M. Carry , A. K. Nguyen , G. R. Merritt , et al., “Prevalence of Persistent Median Arteries in the Pediatric Population on Ultrasonography,” Journal of Ultrasound in Medicine 37 (2018): 2235–2242, 10.1002/jum.14576. 29480530 PMC6109622

[14] S. Arnauw and G. De Wachter , “Thrombosis of Persistent Median Artery as a Cause of Carpal Tunnel Syndrome,” Acta Orthopaedica Belgica 87, no. 3 (2021): 529–532. 34808728

[15] J. Sheridan , G. Waslewski , and D. Sheridan , “Surgical Excision of a Thrombosed Persistent Median Artery in a Professional Hockey Player,” Journal of Hand Surgery 47, no. 3 (2022): 292.e1–292.e4, 10.1016/j.jhsa.2021.01.002. 33726933

[16] A. Baskar, D. S. Pujitha, P. K. Ravi, V. Indiran , “Thrombosis of the Persistent Median Artery: A Rare Cause of Acute Carpal Tunnel Syndrome,” National Journal of Clinical Anatomy 14 (2025): 41–43, 10.4103/NJCA.NJCA_169_24.

[17] K. Osiak , P. Elnazir , A. Mazurek , and A. Pasternak , “Prevalence of the Persistent Median Artery in Patients Undergoing Surgical Open Carpal Tunnel Release: A Case Series,” Translational Research in Anatomy 23 (2021): 100113, 10.1016/j.tria.2021.100113.

[18] E. M. Gassner , M. Schocke , S. Peer , A. Schwabegger , W. Jaschke , and G. Bodner , “Persistent Median Artery in the Carpal Tunnel,” Journal of Ultrasound in Medicine 21 (2002): 455–461, 10.7863/jum.2002.21.4.455. 11934102

